# Whole blood trace element and toxic metal concentration in dogs with idiopathic epilepsy and healthy dogs: A case-control study

**DOI:** 10.3389/fvets.2022.1066851

**Published:** 2023-01-04

**Authors:** Sarah Rosendahl, Johanna Anturaniemi, Tiina-Kaisa Kukko-Lukjanov, Kristiina A. Vuori, Robin Moore, Manal Hemida, Anne Muhle, Anna Hielm-Björkman

**Affiliations:** ^1^Department of Equine and Small Animal Medicine, Faculty of Veterinary Medicine, University of Helsinki, Helsinki, Finland; ^2^Department of Veterinary Biosciences, Faculty of Veterinary Medicine, University of Helsinki, Helsinki, Finland; ^3^Neurology Services, Evidensia Espoo Animal Hospital, Espoo, Finland

**Keywords:** canine, idiopathic epilepsy, trace elements, toxic metals, copper, selenium, chromium, arsenic

## Abstract

**Background:**

Idiopathic epilepsy (IE) is the most common neurological disease in dogs. Multiple genes and environmental factors interact to cause clinical signs, although the pathogenesis remains poorly understood. Extensive evidence from recent decades shows that trace elements play a role in epilepsy in humans, and recently it was shown for the first time that also dogs with IE have altered trace element status. On the other hand, toxic metals may cause seizures but research on their role in canine IE is lacking. Therefore, we aimed to investigate trace element and toxic metal concentrations in whole blood from dogs that had been diagnosed with IE and compare them to those of healthy dogs.

**Materials and methods:**

Whole blood concentrations of trace elements (selenium, zinc, copper, manganese, iron, and chromium) and toxic metals (arsenic, cadmium, mercury, and lead) were analyzed from 19 dogs that had been diagnosed with IE by board-certified neurologists and 19 healthy control dogs using inductively coupled plasma mass spectrometry. The concentrations in study and control group were compared using the Mann-Whitney U test.

**Results:**

Dogs diagnosed with IE had significantly higher blood copper concentration (*P* = 0.007), higher copper/zinc ratio (*P* = 0.04), and higher selenium concentration (*P* < 0.001), as well as lower chromium concentration (*P* = 0.01) when compared to healthy dogs. Treatment of IE with potassium bromide was associated with a significant elevation in blood arsenic concentration (*P* = 0.01).

**Conclusion:**

In conclusion, the present results support the role of altered trace element status in dogs diagnosed with IE and suggest that copper, selenium, and chromium may be involved in the pathogenesis of canine epilepsy or seizures. The results also suggest that potassium bromide may alter arsenic metabolism in dogs.

## 1. Introduction

Epilepsy, which is the dog's most common neurological disorder, is a complex brain dysfunction where the affected dog has a predisposition to generate spontaneous epileptic seizures (1). The etiology of epileptic seizures is currently classified into three groups according to the International Veterinary Epilepsy Task Force: (1) reactive seizures that occur due to metabolic disorders or exposure to toxins (e.g., toxic metals), (2) structural epilepsy which is caused by an identifiable intracranial pathology, and (3) idiopathic epilepsy (IE), which is the most diagnosed form of epilepsy. In IE, genetic factors (either confirmed or suspected) play a role, making certain breeds more prone to epilepsy than others (2, 3), although the underlying cause of seizures often remains unknown (2, 4, 5). The nomenclature in human epilepsy differs from that in dogs. The International League Against Epilepsy classifies epileptic syndromes into six etiologic groups: structural, genetic, infectious, metabolic, immune, and unknown (6). Due to many similarities between epilepsy in dogs and humans, the dog has been suggested as a useful model for epilepsy research (7). In both species, the pathogenesis of epilepsy is still poorly understood, and it is considered that multiple genes and environmental triggers interact to cause clinical signs (2). Epilepsy is a disorder that lowers the quality of life for both the dog and its owner, considering that as much as one-third of epileptic dogs remain unresponsive to antiseizure drug (ASD) treatment (8). In addition, canine epilepsy is associated with behavioral changes (9) and shortened life expectancy (10).

Trace elements are required for neurotransmission, enzyme activities, mitochondrial function, myelination of nerves, and formation of synapses. Imbalanced trace element status has been associated with neurodegeneration, inflammation, and oxidative stress, which all in turn may contribute to neurological diseases and behavioral changes (11). During the last decades, it has become clear that altered trace element status plays a role in human epilepsy (12–18), and in 2019, Vitale et al. (19) reported that also dogs with IE have trace element imbalances. In their study, high serum concentrations of copper (Cu), zinc (Zn), selenium (Se), and manganese (Mn) were found, whereby these trace elements were suggested to play a role in the pathophysiology and/or treatment of epilepsy in dogs. Toxic metal status on the other hand, has not been previously studied in dogs with IE, although there is evidence of chronic toxic metal exposure mimicking IE in dogs (20). Chronic exposure to toxic metals can disrupt neurological function, interfere with the absorption and metabolism of trace elements, and increase oxidative stress (21).

Trace elements and toxic metals can be measured from various loci such as serum, blood, urine, or hair. Whole blood has been considered to give a better reflection of long-term dietary Se intake, as Se is present in a considerable amount in red blood cells, where it has an approximate half-life of 120 days (22). Furthermore, certain intracellular trace elements such as Mn and iron (Fe) may show falsely high values in serum, as hemolysis of the samples may release these trace elements from the blood cells (23). In addition, toxic metals such as lead (Pb) are commonly measured from whole blood, as more than 90% of Pb is bound to red blood cells after absorption (24).

Therefore, the present study used whole blood for measuring trace element and toxic metal status in dogs that had been diagnosed with IE by a small animal board-certified neurologist and healthy control dogs consuming dry or mixed diets. The study aim was to evaluate the potential role of trace elements and/or toxic metals in canine epilepsy.

## 2. Materials and methods

### 2.1. Animals and study design

This case-control study included epileptic and healthy companion dogs living in their home-environment in Finland. Dogs diagnosed with IE were recruited among the regular clients of board-certified neurologists at two small animal veterinary hospitals, Helsinki University Animal Hospital (HUAH) and Evidensia Espoo Animal Hospital, to give blood samples for trace element and toxic metal analysis. The minimum criteria used for IE diagnosis were a history of recurrent epileptic seizures (minimum two), the first seizure occurring between 6 months and 6 years of age as suggested by the International Veterinary Epilepsy Task Force (5), an unremarkable interictal clinical and neurological examination, and an unremarkable complete blood cell count and serum biochemistry profile. In addition to these minimum criteria, several dogs' diagnostic workup also included additional tests. These are presented in Table 1. None of the dogs had brain histopathology performed. The study included a variety of breeds, both breeds that are considered epilepsy prone according to current literature (2, 3) and breeds that are not considered epilepsy prone. Dogs older than 3 years of age that had no current or previous signs of epilepsy or other neurological disease and unremarkable clinical examination, complete blood count, and serum biochemistry profile served as controls. These dogs were from our previous study on hair and blood elements in healthy dogs where we recruited equal numbers of dogs eating dry and raw food to study the effect of diet on trace element status (25). Therefore, the raw fed dogs were excluded from both groups in this study. Exclusion criteria for both study and control groups were pregnancy and lactation, and for the control group being younger than 3 years of age to exclude dogs that possibly might develop epilepsy later. All dog owners were asked to provide information about the dog's health status and feeding in an online questionnaire, and for epileptic dogs, detailed questions about their epilepsy were also asked. The Animal Experiment Board in Finland (ELLA) (permit number: ESAVI/452/2020) approved the study protocol. All dog owners signed a written consent form.

**Table 1:** Tests included in the diagnostic workup of the epileptic dogs (*N* = 19).

**Test**	**Number of dogs that had the test (*n*)**
Complete blood cell count^a^, ^f^	19
Basic serum biochemistry^b^, ^f^	19
Additional blood testing^c^	13
Bile acid stimulation test	12
MRI	8
Vector-borne pathogens^d^	5
Blood ammonia	2
Urinalysis	2
Full thyroid panel^e^	1
CFS analysis	1
EEG	1

MRI, magnetic resonance imaging; CFS, cerebrospinal fluid; EEG, electroencephalogram.

a Leucocytes, erythrocytes, hemoglobin, hematocrit, mean cell volume, mean cell hemoglobin, mean corpuscular hemoglobin concentration, and thrombocytes.

b Alkaline phosphatase, alanine aminotransferase, albumin, total bilirubin, phosphate, glucose, potassium, sodium, calcium, cholesterol, creatinine, protein, and urea.

c Total thyroxine, symmetric dimethylarginine, chloride, gamma glutamyl transferase, aspartate aminotransferase, glutamate dehydrogenase, globulin, a-amylase, lipase, fructosamine, muscle creatine kinase, magnesium, triglycerides, c-reactive protein, reticulocytes, basophils, eosinophils, segmented neutrophils, lymphocytes, and monocytes.

d Anaplasma, Lyme disease, Ehrlichia, and heartworm.

e thyroxine, free thyroxine, thyrotropin, and thyroxine/thyrotropin.

f included in the minimum criteria for IE diagnosis.

### 2.2. Whole blood trace element and toxic metal analysis

All samples were fasting samples. For analysis of trace elements and toxic metals, blood was collected from the cephalic vein into 6 ml NH Trace Elements Sodium Heparin tubes and then divided into 1.5 ml Eppendorf tubes and stored at −20°C until analyzed 6–12 months later. Analysis of whole blood Se, Zn, Cu, Mn, Fe, chromium (Cr), arsenic (As), cadmium (Cd), mercury (Hg), and Pb was performed at the Department of Environmental Sciences, Jožef Stefan Institute (Ljubljana, Slovenia). Altogether 0.3 g of whole blood samples was transferred into pre-cleaned teflon digestion vials. Samples were digested by 0.5 ml of 65% nitric acid (HNO_3_, 65% Suprapur^®^ for trace analysis, Supelco, Merck KGaA, Darmstadt, Germany) in a microwave system (ULTRAWAVE, Single Reaction Chamber Microwave Digestion System, MILESTONE, Italy) using the following program: (1) 20 min temperature rise to 240°C, (2) kept 12 min at 240°C and max 100 bar. The remaining solutions were transferred into measuring tubes and further diluted to 5 ml with deionized water (18.2MΩ cm) obtained using Milli-Q system (Merck, Millipore, Watertown, MA, USA). Prepared solutions were measured by Triple Quadrupole Inductively Coupled Plasma Mass Spectrometry (ICP-QQQ, Agilent 8800, California, USA). The Inductively Coupled Plasma Mass Spectrometry (ICP-MS) instrument operation parameters are presented in Table 2. Isotopes monitored were: ^52^Cr, ^55^Mn, ^57^Fe, ^63^Cu, ^66^Zn, ^75^As, ^78^Se, ^114^Cd, ^202^Hg, and ^208^Pb. External calibration was used for quantification. For calibration, Hg single element standard NIST 3133 and multi standard Periodic table mix 1 for ICP (TraceCERT^®^, Sigma-Aldrich, 33 elements) were used. Accuracy of results was checked using two reference materials: Seronorm Whole blood Level 1 (lot: 1702821; SERO) and Level 2 (lot: 1702825; SERO). The laboratory method quality control results are presented in Table 3 and the validation parameters of the ICP-MS method are presented in Table 4. Limits of detection (LOD) calculated as 3 times standard deviation of several blank samples were: 1.5 ng/g for Cr, 0.4 ng/g for Mn, 40 ng/g for Fe, 1.5 ng/g for Cu, 150 ng/g for Zn, 0.2 ng/g for As, 1 ng/g for Se, 0.02 ng/g for Cd, 0.04 ng/g for Hg, and 0.4 ng/g for Pb.

**Table 2:** ICP-MS instrument operation parameters.

**Parameter**	**Value**
Nebulizer	MicroMist
Spray chamber	Scott double-pass
RF Power	1550 W
Plasma gas flow	15 L min^−1^
Carrier gas	0.95 L min^−1^
Makeup gas	0.10 L min^−1^
Sampling depth	8.0 mm
Sample uptake rate	0.1 rps
Sampling and skimmer cones	Nickel
Isotopes of internal standards	45Sc, ^89^Y,^103^Rh, ^157^Gd
Cell gases at ORS Isotopes of measured elements	Helium, hydrogen or oxygen 52Cr, ^55^Mn, ^57^Fe, ^63^Cu, ^66^Zn, ^75^As, ^78^Se, ^114^Cd, ^202^Hg, and ^208^Pb.

RF, radio frequency; ORS, octopole reaction system.

**Table 3:** Results obtained for reference materials used for quality control of whole blood element analysis.

**Element (ng/g)**	**Seronorm trace elements whole blood L-1; lot: 1702821**				**Seronorm trace elements whole blood L-2; lot: 1702825**			

	**Avg** **(*****N** =* **16)**	**Rsd (%)**	**Reference value**	**Reference range**	**Avg** **(*****N** =* **16)**	**Rsd (%)**	**Reference value**	**Reference range**
Fe	347945	5.4	357000	NA	353431	5.6	350000	NA
Cu	568	5.0	640	590-700	875	5.6	980	890-1060
Mn	18.7	4.5	19.7	18.1–21.3	23.9	6.4	24.2	22.2–26.1
Zn	4327	8.2	4600	3800–5300	5823	3.8	5800	4800–6800
Se	66.0	6.1	69	54-84	137	4.3	144	113–175
Cr	<LOD	NA	0.77	0.61–0.92	11.78	8.8	10.1	8.0–12.0
Pb	9.72	2.5	10	7.9–12	290	1.4	303	272–334
Hg	1.50	7.9	1.57	1.25–1.88	14.9	2.5	16.6	13.3–20.0
Cd	0.32	20.5	0.28	0.23–0.34	5.21	3.5	5.1	4.1–6.1
As	2.12	8.6	2.1	1.7–2.5	11.7	3.7	12.2	9.8–14.7

avg, arithmetic mean; rsd, relative standard deviation; NA, not available; LOD, limit of detection.

**Table 4:** Validation parameters of the ICP-MS method aimed at determining trace element and toxic metal concentrations in whole blood.

**Element**	**Limit of detection (ng/g)**	**Limits of quantification (ng/g)**	**Maximum linear concentrations**	**Recovery (%)**	**Precision coefficient of variatio*n* (%)**
Fe	40	130	250	99	5.5
Cu	1.5	5	250	89	5.3
Mn	0.4	1.3	250	97	5.5
Zn	150	500	250	97	6.0
Se	1	3	250	95	5.2
Cr	1.5	5	250	116	8.8
Pb	0.4	1.3	250	97	2.0
Hg	0.04	0.13	10	93	5.2
Cd	0.02	0.07	250	108	12
As	0.2	0.7	250	98	6.1

### 2.3. Statistical analysis

SPSS for Windows (version 27; IBM SPSS Statistics) was used for all analyses. Nondetectable element concentrations were assigned a value of LOD divided by the square root of 2. The normality of the data was assessed using the Shapiro-Wilk test. Study and control group characteristics were compared using independent sample *t*-test for age and weight, and chi-square test for sex, diet, living environment, and drinking water. Trace and toxic element concentrations in study and control groups were compared using the Mann–Whitney *U-*test. Statistical significance was set at *P* < 0.05 in all analyses. To assess the sample size needed to detect a significant difference between two means a power calculation was performed based on human epilepsy studies (13, 17), as there were no relevant canine studies at the planning stage of this study. Based on this calculation the number of animals needed would be 30–34 in each group.

## 3. Results

### 3.1. Animals

Blood samples were collected from a total of 21 dogs with IE and 33 controls. As sampling criteria differed between the cases and controls regarding raw feeding, all raw fed dogs were excluded from the data (2 epileptic and 14 control dogs) to avoid a possible confounding effect of diet when comparing trace element and toxic metal concentrations between groups. Thus, blood samples were analyzed from a total of 19 dogs with IE and 19 controls. The epileptic group included 11 males and nine females (mean age 5.2 years, range 1.6–10.7) and the control group included nine males and 10 females (mean age 6.0 years, range 3.0–12.1). The groups were, by chance alone, statistically very similar regarding age, weight, sex, diet, living environment, and drinking water (Table 5). Both groups consisted of a variety of breeds, and most epileptic dogs (18/19) received one or more ASDs, the most common being phenobarbital and potassium bromide (KBr). Detailed information about each dog's characteristics (breed, sex, age, and weight) and diet, as well as for epilepsy dogs, data on the diagnostic workup and ASD treatment, is presented in Supplementary Table 1. The table was made using owner-provided questionnaire data and/or data collected from EEAH/HUAH data bases. As we wanted to see if genetic factors affected our results, we also divided the epileptic dogs into two groups: one group with breeds that are considered epilepsy prone and/or having a reported family history of epilepsy (*n* = 11) and one group with other epileptic dogs (*n* = 8). This classification of breeds can also be seen in Supplementary Table 1.

**Table 5:** Characteristics and statistical comparison of the study and control groups.

	**Epileptic (*n =* 19)**	**Healthy (*n =* 19)**	**P**
Age, years, mean (min–max)^a^	5.2 (1.6–10.7)	6.0 (3.0–12.1)	0.313
Weight, kg, mean (min-max)^a^	25.7 (8.5–65)	22.8 (10–50.1)	0.468
Sex, *n* (%)^b^			0.746
Male	11 (57.9)	9 (47.4)	
Female	8 (42.1)	10 (52.6)	
Diet, *n* (%)^b^			
Dry	12 (63.2)	13 (68.4)	1.000
Mixed^c^	7 (36.8)	6 (31.6)	
Living environment, *n* (%)^b^			0.476
Rural	12 (63.2)	15 (78.9)	
Urban	7 (36.8)	4 (21.1)	
Drinking water, *n* (%)^b^			1.000
Tap water	16 (84.2)	17 (89.5)	
Well water	3 (15.8)	2 (10.5)	

a Independent samples t-test.

b Chi-Square test.

c Mix of dry, raw, home-cooked and/or canned food.

### 3.2. Blood trace element and toxic metal concentrations

**Figure 1:**
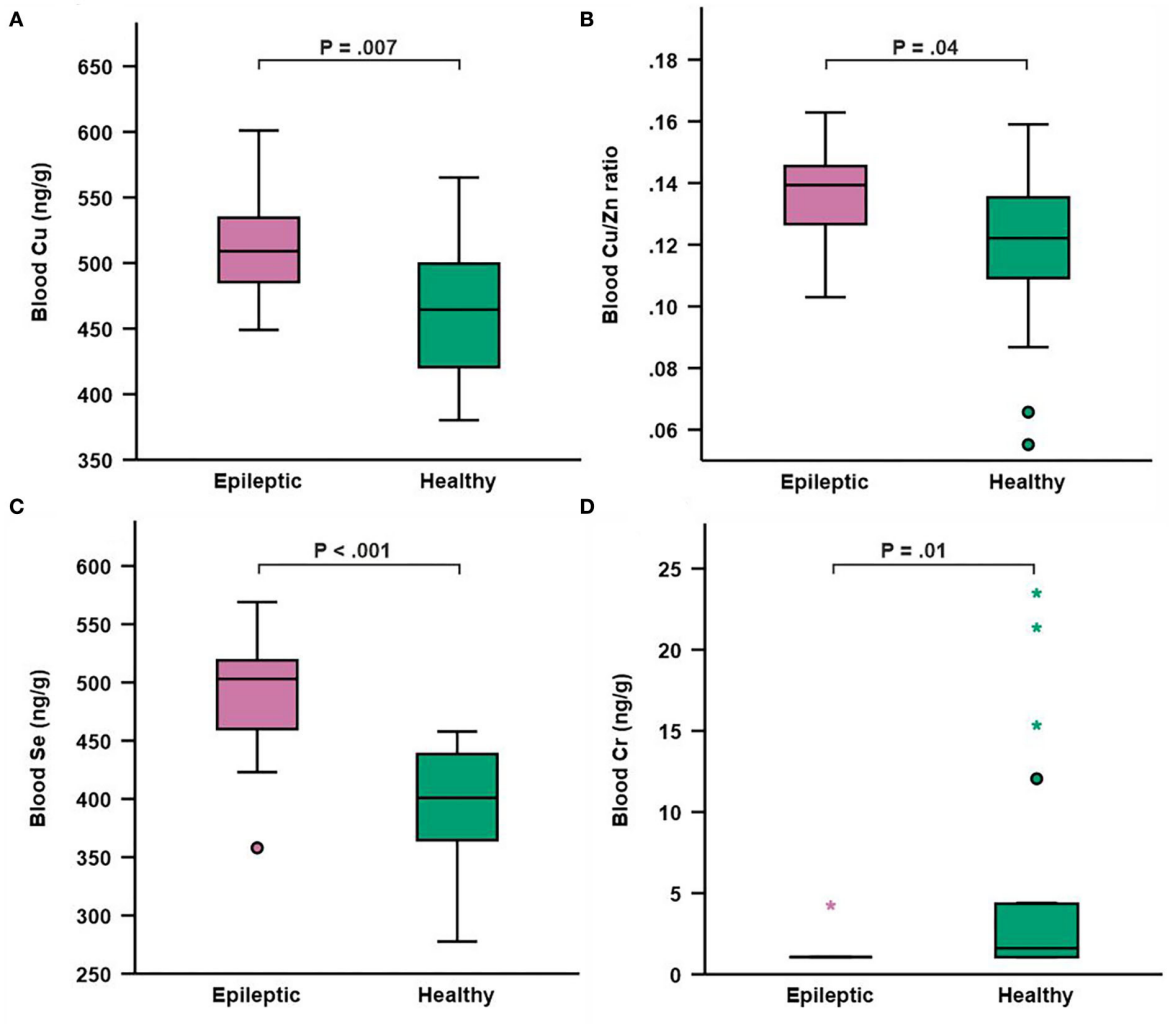
Box and whisker plot depicting whole blood concentrations of copper **(A)** copper/zinc ratio **(B)** selenium **(C)** and chromium **(D)** in epileptic (*n* = 19) and healthy (*n* = 19) dogs. Boxes represent the interquartile range from the 25th to 75th percentile, while the horizontal lines in each box represent the median. Whiskers indicate the range of the data, excluding outliers, which are indicated by circles (o) when >1.5 times the IQR and by asterisks (*) when >3 times the IQR.

Mean whole blood trace element and toxic metal concentrations in study and control groups are presented in Table 6. Epileptic dogs had significantly higher blood Cu concentration compared to healthy dogs (*P* = 0.007; Table 6, Figure 1A). We calculated the blood Cu/Zn ratio and found that also this was significantly higher in epileptic compared to healthy dogs (*P* = 0.04; Table 6, Figure 1B). Blood Se concentration was also significantly higher in epileptic compared to healthy dogs (*P* < 0.001; Table 6, Figure 1C). Blood Cr concentration was significantly lower in epileptic compared to healthy dogs (*P* = 0.011; Table 6, Figure 1D). Among the epileptic dogs, the majority (18/19 or 94.7 %) had Cr levels that were below the LOD, while only 9 out of 19 (47.4 %) of the healthy dogs presented with such levels. For Zn, Mn, Fe, As, Cd, Hg, and Pb, no significant differences in blood concentrations were found between epileptic and healthy dogs. However, we found that dogs receiving KBr (*n* = 6) had significantly higher concentration of As in the blood (5.35 μg/g ± 6.52; 1.04–18.10) when compared to epileptic dogs treated with other ASDs (*n* = 12) (1.47 μg/g ± 1.50; 0.50–5.75) (*P* = 0.03) or to healthy dogs (*n* = 19) (1.33 ± 1.20, 0.12–4.27) (*P* = 0.01). Regarding Hg, we identified an extreme outlier among the epileptic dogs with a blood Hg concentration that was almost 16-fold higher than the mean concentration in healthy dogs. Genetic factors had no significant effect on the results (data not shown).

**Table 6:** Whole blood trace element and toxic metal concentrations in epileptic and healthy dogs.

**Blood element** **(ng/g)** **Reference values from literature**	**Epileptic (*n =* 19)** **Mean ±SD (min–max)**	**Healthy (*n =* 19)** **Mean ±SD (min–max)**	**P**
Cu 483.79 ± 59.04 (349.70–614.20)^a^	511.95 ± 40.44 (449–601)	463.20 ± 55.17 (380–565)	0.007^**^
Zn 3685.56 ± 542.69 (2850–5630)^a^	3780.53 ± 341.10 (3120–4360)	4158.42 ± 1380.98 (2990–8420)	0.86
Cu/Zn ratio	0.14 ± 0.02 (0.10–0.16)	0.12 ± 0.03 (0.06–0.16)	0.04^*^
Se 388.14 ± 40.95 (310.10–476.60)^a^	488.53 ± 47.69 (358–569)	393.90 ± 51.89 (278–458)	<0.001^***^
Cr 3.67 ± 6.07 (1.06–24.83)^a^	1.23 ± 0.73 (1.06–4.25)	5.16 ± 7.25 (1.06–23.49)	0.01^*^
Mn 30.84 ± 9.78 (14.50–59.50)^a^	34.64 ± 11.75 (18.90–60.50)	36.19 ± 9.62 (21.24–59.53)	0.51
Fe 605382.98 ± 62129.09 (486000–742000)^a^	625368.42 ± 55588.98 (510000–780000)	599526.32 ± 62726.36 (508000–713000)	0.12
As 1.15 ± 1.09 (0.04–4.30)^a^	2.65 ± 4.09 (0.50–18.10)	1.33 ± 1.20 (0.12–4.27)	0.30
Cd 0.03 ± 0.01 (0.01–0.06)^a^	0.02 ± 0.02 (0.01–0.09)	0.03 ± 0.03 (0.01–0.13)	0.10
Hg 0.53 ± 0.35 (0.12–1.61)^a^	0.94 ± 1.58 (0.11–7.29)	0.46 ± 0.33 (0.12–1.14)	0.18
Pb 3.50 ± 2.72 (0.28–12.37)^a^	2.17 ± 1.80 (0.32–6.51)	2.88 ± 2.07 (0.28–8.04)	0.22

^*^Correlation is significant at the 0.05 level, ^**^at the 0.01 level and, ^***^at the 0.001 level (all 2-tailed). ^a^Rosendahl et al. (25), mean± SD (min-max).

## 4. Discussion

This study showed that dogs with IE had higher blood concentration of Cu and Se, as well as a higher blood Cu/Zn ratio, and a lower blood concentration of Cr, compared to healthy dogs. Potassium bromide was associated with an elevated blood As concentration.

Blood Cu concentration was significantly higher in epileptic compared to healthy dogs, which is consistent with the results by Vitale et al. (19). In their study, the elevated Cu levels were associated with phenobarbital treatment, which may raise Cu levels by increasing hepatic synthesis of ceruloplasmin (26). Most of the epileptic dogs included in our study were treated with phenobarbital, and thus this may have affected our results as well. However, several human studies support our finding of higher Cu levels in epileptic patients. Prasad et al. (27) studied 200 patients with idiopathic generalized epilepsy and discovered that they had a significantly higher serum Cu concentration compared to 200 healthy controls. In another study, children with idiopathic seizures were found to have significantly elevated serum Cu concentration compared to healthy children (28). Likewise, Ilhan et al. (16) found higher serum Cu concentration in epileptic patients compared to healthy controls. Certain ASDs such as phenytoin has been shown to increase serum Cu levels in humans (18), although Cu levels seem to be increased also in drug-naïve epileptic patients (29). Unfortunately, reference values for blood Cu in dogs have not been established. However, the mean blood Se concentration of the epileptic dogs in the current study (511.95 ng/g) was higher than that reported in our previous study on 50 healthy dogs (483.79 ng/g) (25) and in another study by Panda et al. (30) (480 ng/g), although none of the epileptic dogs had Cu concentrations that were outside the range that were reported in these two studies. Our study also found that the Cu/Zn ratio, which in humans has been regarded as clinically more important than the concentrations of Cu or Zn alone (31), was significantly higher in epileptic compared to healthy dogs, which is consistent with what has been reported in serum of children with idiopathic seizures (28). Children with febrile seizures have also been found to have increased serum Cu concentrations, together with decreased Zn concentration and increased oxidative stress (32). However, as there are no previous studies on the Cu/Zn ratio in dogs, this finding needs to be interpreted with caution. Being involved in neurotransmitter synthesis, modulation of synaptic activity, and myelination of nerves, Cu plays an important role in brain and nervous system health, with both excess and deficiency having potentially harmful effects on neuronal functions (33). In rats, even low doses of injected Cu may inhibit the enzymes Mg-ATPase and Na, K-ATPase, leading to disturbed Na and K homeostasis and inducing seizures (34), although it is unknown if similar effects could occur in dogs. Furthermore, increased Cu can alter neuronal excitability and synaptic communication by affecting N-methyl-D-aspartate (NMDA) receptors and voltage-gated calcium channels (35). Excess Cu has been associated with oxidative stress and neuroinflammation, whereas Zn has antioxidant and anti-inflammatory properties (31) and thus, the Cu/Zn ratio has been considered a marker of oxidative stress and inflammation (36, 37), of which the latter has been suggested to be involved in the pathophysiology of canine IE (38). The reason for higher blood Cu concentration in the epileptic dogs in our study remains unclear. A study from 2018 reported that hepatic Cu concentrations have increased over time in dogs, which was suggested to be a result of the increased usage of Cu water pipes and raising Cu supplementation recommendations for commercial dog food (39). Thus, it is possible that overexposure to Cu from water and diet is a triggering factor involved in canine IE. However, further research is needed to clarify the role of Cu and/or Zn in canine IE.

Blood Se concentration was significantly higher in epileptic compared to healthy dogs. Our finding is in line with the study by Vitale et al. (19) where epileptic dogs had higher serum Se concentration compared to healthy dogs, although the difference was only significant for those receiving ASD treatment. Selenium has been clearly associated with epilepsy also in humans. Interestingly, according to a recent meta-analysis, a majority of human studies have found Se concentration in epileptic patients to be lower than in healthy controls (40). However, a recent study found that serum Se concentration was significantly higher in epileptic patients compared to controls (41), which is consistent with our results. We have also recently shown that dogs with IE have significantly higher Se concentration in the hair, even if they are not receiving ASD treatment (manuscript in revision). In humans, the lower Se levels in epileptic patients have been associated with increased oxidative stress, as Se is an essential part of the antioxidant enzyme glutathione peroxidase (15, 17). However, Se has a narrow margin of safety, with the difference between adequate and potentially harmful concentrations in the diet being quite low, and in excess, Se can have pro-oxidant or other adverse effects (42, 43). In dogs, chronic Se poisoning from food, i.e., selenosis, is associated with symptoms such as anorexia, emaciation, growth retardation, ascites, anemia, coarse and loose hair, and eventual death (44). Although the dogs in our study did not show apparent signs of selenosis, non-specific symptoms such as hair changes might have been overlooked. As reference values for Se in whole blood are lacking for dogs (44), it is difficult to draw firm conclusions. However, the mean blood Se concentration in epileptic dogs in the current study (488.53 ng/g) is clearly higher than that reported in our previous study on 50 clinically healthy dogs (388.14 ng/g). Twelve out of the 19 epileptic dogs also had blood Se concentrations that were higher than the maximum concentration reported in that study (25). As dogs commonly eat commercial diets with added Se, they are very unlikely to become deficient. Instead, there have been several reports of excess Se in commercial dog foods (45–47). Dogs as carnivores also retain more Se and maintain higher serum Se concentration compared to herbivore and omnivore species, and it has been suggested that also other aspects of Se metabolism could differ in dogs (44), although the research in this area is limited. In human studies, Se overexposure has recently been associated with adverse effects such as altered glucose metabolism and diabetes (48), and it is possible that similar, yet unknown, effects exist in dogs. It has been reported that Se has an antagonistic relationship with Cr (49), and in a study on rats, high intake of Se depleted Cr levels in serum and liver and led to increased oxidative stress and hepatic insulin resistance. Activation of selenoproteins together with Cr deficiency also resulted in a common metabolic downstream effect of enhanced lipolysis in adipose tissue and free fatty acid accumulation in the liver (50). Interestingly, we found very low blood Cr levels in the epileptic dogs in our study. In summary, Se appears to play a role in epilepsy in both humans and dogs: in humans, low Se has been associated with increased oxidative stress, which in turn has been strongly implicated in seizure disorders (15), while in dogs, further research is needed to clarify the mechanisms by which high Se is potentially related to the epilepsy pathogenesis.

Blood Cr concentration was significantly lower in epileptic compared to healthy dogs, with all except one epileptic dog having Cr levels below the limit of detection. Chromium is an essential trace element involved in carbohydrate and lipid metabolism. By enhancing the effects of insulin, improving insulin binding to cells, and increasing insulin receptor numbers and phosphorylation, Cr plays a key role in maintaining a normal glucose metabolism and insulin sensitivity and deficiency has been associated with insulin resistance, glucose intolerance, and hyperglycemia (51). Supplementation with Cr has been proven successful in improving glucose metabolism in humans with type 2 diabetes (52), although studies in dogs have failed to reveal any clear benefits (53). Impaired glucose metabolism has been found in epileptic brain areas, and the successful management of epilepsy in both humans and dogs by using ketogenic or medium chain triglyceride (MCT) diets indicates that certain types of epilepsy may be caused by a dysfunctional energy metabolism (54). According to Vianna et al. (55), patients with refractory epilepsy often suffer from glucose intolerance as a result of undiagnosed metabolic disturbances. In line with our finding in dogs, drug-naïve epileptic children were found to have significantly lower Cr concentrations in serum compared to healthy children. The serum Cr concentration in these children was also negatively correlated with the serum glucose concentration, which could not be seen in the healthy children (56). Research on Cr status in dogs is limited to a few studies on dogs with cancer, showing that they have a decreased Cr concentration in serum (57) and hair (58) compared to healthy dogs. It was suggested that the altered carbohydrate metabolism observed in dogs with cancer could be related to Cr deficiency (57). Both Se and Cr have been considered key metals involved in the pathogenesis of glucometabolic disorders (59). Future studies should investigate whether the altered concentrations of blood Se and Cr in epileptic dogs could be related to an impaired glucose metabolism that in turn may be causing or contributing to reactive seizures that clinically mimic IE. As we saw a trend towards fewer epileptic dogs among raw diet-fed dogs in our previous study on hair elements (manuscript in revision), it would be important to look at the effect of raw feeding in future epilepsy studies. Raw diets, which are mainly ketogenic (low in carbohydrates and high in fat) (60), provide other brain fuels than glucose (e.g., ketone bodies), which have shown beneficial effects for the management of epilepsy in both humans and dogs (54, 61). However, our efforts to recruit adequate numbers of raw fed epileptic dogs for our studies have been unsuccessful, so far.

Blood As concentration was significantly higher in dogs treated with KBr compared to those treated with other ASDs or to healthy dogs. We have recently seen this also in the hair of dogs with IE (manuscript in revision). The reason for this finding is unclear. It could be related to kidney excretion, since both bromide (62) and As (63) are excreted through the kidneys. However, also other factors, such as methylation, may affect the excretion of As from the body (63). Because of the low number of dogs treated with KBr in this study these results should be interpreted with caution.

The extreme outlier for blood Hg concentration among the epileptic dogs was eating a mixed diet consisting of 70% cooked saithe, 30% salmon-based raw food, as well as daily tuna treats. Large fish species such as tuna are a known source of methylmercury (64), which is a potent neurotoxin that has been associated with epileptic seizures and increased seizure susceptibility (65). Although it is unclear if Hg played a role in this dog's epilepsy, future studies should assess Hg burden in a larger study population to see if Hg toxicity may play a role in seizure etiology in individual epileptic dogs.

Even though the dogs in our study were diagnosed with IE by board-certified small animal neurologists, they showed trace element imbalances that might be suggestive of metabolic alterations. Therefore, the pathogenesis of the seizures in the IE-diagnosed dogs in the current study could be reactive or metabolic in nature. Based on these findings, it could be beneficial to alter the classification of canine epilepsy so that it is in line with the human epilepsy classification.

The main limitation of our study was the small sample size. Due to our sample collection occurring during COVID restrictions, we were not able to recruit more dogs. In addition, exclusion criteria used further decreased our sample size. Another limitation was that our study included only one untreated epileptic dog. Future studies should include a larger sample size, including a group of drug-naïve dogs to get a better understanding of how ASDs affect trace element and toxic metal status. Finally, the study did not specify epileptic syndromes. For example, seizure strength affects body metabolism and could thus potentially also affect trace element metabolism. Future studies should also assess the dietary intake of the studied elements to clarify whether the observed alterations are related to dietary intake or to metabolic or other factors.

## 5. Conclusion

In conclusion, this study showed that dogs with IE have higher blood Cu, Cu/Zn ratio, and Se, as well as lower blood Cr compared to healthy dogs, reinforcing the role of altered trace element status in dogs diagnosed with IE. The study also showed that KBr treatment may alter As metabolism in dogs. Our findings highlight the difficulty of using the current canine classification of epilepsy or seizures. However, these results need to be confirmed through studies with larger sample size, and future studies should aim to elucidate the mechanisms by which trace elements may be involved in the pathogenesis of canine epilepsy or seizures.

## Data availability statement

The raw data supporting the conclusions of this article will be made available by the authors, without undue reservation.

## Ethics statement

The animal study was reviewed and approved by the Animal Experiment Board in Finland (ELLA) (permit number: ESAVI/452/2020). Written informed consent was obtained from the owners for the participation of their animals in this study.

## Author contributions

SR, AH-B, T-KK-L, and JA designed the research. SR, AH-B, and AM collected the samples. SR, AH-B, and KV performed data and statistical analyses. SR and T-KK-L wrote the manuscript. AH-B, JA, KV, RM, and MH contributed to analysis and interpretation of the data and revised the manuscript. All authors read and approved the final manuscript.
