# Supplementary material for: Whole blood trace element and toxic metal concentration in dogs with idiopathic epilepsy and healthy dogs: A case-control study
Source: Front Vet Sci. 2023 Jan 4;9:1066851. doi: 10.3389/fvets.2022.1066851 (PMC9845892; doi:10.3389/fvets.2022.1066851)
Supplement: Supplementary file 1 [file Table_1.pdf]

Supplementary Material

Supplementary Table 1 Detailed information about study (n=19) and control dogs (n=19)

Dog nr	Group	Breed	Genetic factors	Age (y)	Sex	Weight (kg)	Diet	Age at seizure onset (y)	Diagnostic workup for epileptic dogs								ASD treatment
									Place of diagnosis	*Clinical general exam*	*Neurological exam*	*Blood tests*	*MRI*	*EEG*	*CSF*	*Other*	
1	untreated epileptic	Golden Retriever	epilepsy prone breed[a]	3.6	male, neutered	33.5	dry	3.3	EEAH	x	x	x[d,e]					
2	treated epileptic	German Shepherd	epilepsy prone breed	6.1	male, intact	41.0	mixed	2.7	HUAH	x	x	x[c]	x				Phenobarbital
3	treated epileptic	Lagotto Romagnolo	epilepsy prone breed	4.1	male, neutered	20.7	mixed	0.8	HUAH	x	x	x[c]					Phenobarbital
4	treated epileptic	Mixed breed	family history[b]	4.5	male, intact	30.0	dry	1.8	HUAH	x	x	x[c,f]				urinalysis	Phenobarbital, potassium bromide, levetiracetam
5	treated epileptic	Bull Terrier	none	7.8	female, spayed	16.5	mixed	4.0	HUAH	x	x	x[c]	x	x			Phenobarbital, potassium bromide
6	treated epileptic	Australian Terrier	none	1.6	female, spayed	8.5	mixed	0.6	HUAH	x	x	x[c,f]					Imepitoin
7	treated epileptic	Red Irish Setter	none	5.1	female, intact	25.0	mixed	3.8	HUAH	x	x	x[c]	x				Imepitoin
8	treated epileptic	Giant Schnauzer	family history	3.2	male, intact	43.5	dry	2.6	EEAH	x	x	x[d,e,g]	x				Phenobarbital
9	treated epileptic	Saint Bernard	none	4.5	female, intact	65.0	dry	4.1	EEAH	x	x	x[d,e,g]					Phenobarbital, potassium bromide

10	treated epileptic	Flat-coated Retriever	none	5.2	male, neutered	32.5	dry	2.1	EEAH	x	x	x[d,e,g]				urinalysis	Phenobarbital
11	treated epileptic	Soft-coated Wheaten Terrier	none	8.5	female, spayed	19.4	dry	0.5	EEAH	x	x	x[d]	x				Phenobarbital, potassium bromide, levetiracetam
12	treated epileptic	Border Collie	epilepsy prone breed	4.2	male, neutered	18.0	dry	3.0	EEAH	x	x	x[d,e]					Phenobarbital
13	treated epileptic	Hungarian Wirehaired Vizsla	epilepsy prone breed	5.3	male, intact	32.5	dry	4.0	EEAH	x	x	x[d,e]	x				Phenobarbital
14	treated epileptic	Cocker Spaniel	epilepsy prone breed	3.2	female, intact	12.8	dry	2.3	EEAH	x	x	x[d,e,g]	x		x		Phenobarbital, imepitoin
15	treated epileptic	Volpino Italiano	none	10.7	male, intact	10.4	dry	6.6	EEAH	x	x	x[d,e]	x				Phenobarbital, levetiracetam
16	treated epileptic	Australian Labradoodle	family history	3.7	male, neutered	27.1	dry	2.0	EEAH	x	x	x[d,e]					Phenobarbital
17	treated epileptic	Mixed breed	none	5.9	male, neutered	17.7	mixed	0.6	EEAH	x	x	x[d,e,h]					Phenobarbital, potassium bromide, levetiracetam
18	treated epileptic	Australian Shepherd	epilepsy prone breed	3.6	female, intact	24.0	mixed	2.2	EEAH	x	x	x[d,e,g]					Imepitoin
19	treated epileptic	Border Terrier	epilepsy prone breed	8.2	female, intact	10.8	dry	3.0	EEAH	x	x	x[d,e]					Potassium bromide
20	healthy	Australian Shepherd		6.1	male, intact	25.0	dry										
21	healthy	Mixed breed		6.0	male, intact	25.0	dry										

22	healthy	Mixed breed		9.6	male, neutered	22.0	dry										
23	healthy	Norwegian Elkhound		9.3	female, spayed	34.9	dry										
24	healthy	Mixed breed		4.1	male, neutered	21.0	dry										
25	healthy	Mixed breed		6.0	male, neutered	29.0	dry										
26	healthy	German Shepherd		5.2	female, intact	29.0	mixed										
27	healthy	Czech Mountain Dog		4.1	female, spayed	20.0	dry										
28	healthy	Mixed breed		3.7	male, neutered	10.8	mixed										
29	healthy	Australian Kelpie		7.3	male, neutered	23.0	dry										
30	healthy	Pumi		12.1	female, intact	11.5	mixed										
31	healthy	Cavalier King Charles Spaniel		8.5	female, intact	10.0	dry										
32	healthy	Finnish Lapphund		5.4	female, intact	18.0	mixed										
33	healthy	Mixed breed		3.2	female, intact	25.0	mixed										
34	healthy	Bernese Mountain Dog		3.0	female, intact	37.0	dry										
35	healthy	Cocker Spaniel		3.7	female, intact	18.0	mixed										
36	healthy	Mixed breed		6.8	female, spayed	14.3	dry										
37	healthy	Bullmastiff		3.7	male, intact	50.1	dry										
38	healthy	Kromfohrländer		6.1	male, intact	10.0	dry										

ASD, antiseizure drug; CSF, cerebrospinal fluid analysis; EEAH, Evidensia Espoo Animal Hospital; EEG, electroencephalogram; HUAH, Helsinki University Animal Hospital; MRI, magnetic resonance imaging; y, years.

[a]epilepsy prone breed according to literature (ref. 2,3)

[b]family history reported by owner in questionnaire

[c]complete blood cell count (leucocytes, erythrocytes, hemoglobin, hematocrit, mean cell volume, mean cell hemoglobin, mean corpuscular hemoglobin concentration, and thrombocytes) and basic serum biochemistry (alkaline phosphatase, alanine aminotransferase, albumin, total bilirubin, phosphate, glucose, potassium, sodium, calcium, cholesterol, creatinine, protein, and urea)

[d]same as c, but additionally total thyroxine, symmetric dimethylarginine, chloride, gamma glutamyl transferase, aspartate aminotransferase, glutamate dehydrogenase, globulin, a-amylase, lipase, fructosamine, muscle creatine kinase, magnesium, triglycerides, c-reactive protein, reticulocytes, basophils, eosinophils, segmented neutrophils, lymphocytes, and monocytes

[e]bile acid stimulation test

[f]ammonia

[g]vector-borne pathogens (Anaplasma, Lyme disease, Ehrlichia, heartworm)

[h]thyroid profile (thyroxine, free thyroxine, thyrotropin, thyroxine/thyrotropin)
